# Measuring patient activation in the Netherlands: translation and validation of the American short form Patient Activation Measure (PAM13)

**DOI:** 10.1186/1471-2458-12-577

**Published:** 2012-07-31

**Authors:** Jany Rademakers, Jessica Nijman, Lucas van der Hoek, Monique Heijmans, Mieke Rijken

**Affiliations:** 1 NIVEL – Netherlands Institute for Health Services Research, PO Box 1568, 3500, BN, Utrecht, The Netherlands

## Abstract

**Background:**

The American short form Patient Activation Measure (PAM) is a 13-item instrument which assesses patient (or consumer) self-reported knowledge, skills and confidence for self-management of one’s health or chronic condition. In this study the PAM was translated into a Dutch version; psychometric properties of the Dutch version were established and the instrument was validated in a panel of chronically ill patients.

**Methods:**

The translation was done according to WHO guidelines. The PAM 13-Dutch was sent to 4178 members of the Dutch National Panel of people with Chronic illness or Disability (NPCD) in April 2010 (study A) and again to a sub sample of this group (N = 973) in June 2010 (study B). Internal consistency, test-retest reliability and cross-validation with the SBSQ-D (a measure for Health literacy) were computed. The Dutch results were compared to similar Danish and American data.

**Results:**

The psychometric properties of the PAM 13-Dutch were generally good. The level of internal consistency is good (α = 0.88) and item-rest correlations are moderate to strong. The Dutch mean PAM score (61.3) is comparable to the American (61.9) and lower than the Danish (64.2). The test-retest reliability was moderate. The association with Health literacy was weak to moderate.

**Conclusions:**

The PAM-13 Dutch is a reliable instrument to measure patient activation. More research is needed into the validity of the Patient Activation Measure, especially with respect to a more comprehensive measure of Health literacy.

## Background

In 2004 Hibbard et al. developed and tested the Patient Activation Measure (PAM), a 22-item instrument which assesses patient (or consumer) self-reported knowledge, skills and confidence for self-management of one’s health or chronic condition [1]. In 2005 a 13-item Short Form of the PAM was developed, which has the same psychometric properties as the longer version and is both reliable and valid – see Table 1[2]. Both versions of the instrument measure the level of activation of a specific individual. The PAM divides consumers and patients into one of four progressively higher activation levels, which are associated with specific self-care and other health related behaviours. Research has repeatedly shown that a higher score on the PAM is positively associated with various health related behaviours, such as preventive care and lifestyle behaviours, information seeking and use of health information, health outcomes and healthcare use, monitoring and medication adherence, conduct in the patient-provider encounter and self-management [3,4]. These effects have been demonstrated both in a clinical setting with chronically ill patients but also in other populations (e.g. company employees, senior citizens in community centres) [5,6].

**Table 1:** 13 item Patient Activation Measure ¹


1	When all is said and done, I am the person who is responsible for managing my health condition
2	Taking an active role in my own health care is the most important factor in determining my health and ability to function
3	I am confident that I can take actions that will help prevent or minimize some symptoms or problems associated with my health condition
4	I know what each of my prescribed medications do
5	I am confident that I can tell when I need to go get medical care and when I can handle a health problem myself
6	I am confident I can tell my health care provider concerns I have even when he or she does not ask
7	I am confident that I can follow through on medical treatments I need to do at home
8	I understand the nature and causes of my health condition(s)
9	I know the different medical treatment options available for my health condition
10	I have been able to maintain the lifestyle changes for my health that I have made
11	I know how to prevent further problems with my health condition
12	I am confident I can figure out solutions when new situations or problems arise with my health condition
13	I am confident that I can maintain lifestyle changes like diet and exercise even during times of stress

^**1**^ Original version [2].

Though the concept of patient activation has a high face validity and was carefully constructed using experts consensus and patient focus groups [1], an important question remains what construct the instrument is actually measuring. Studies on the construct validity of the PAM predominantly focus on the possible overlap with the concept of health literacy. In some studies the relative contribution of health literacy and patient activation was examined in relation to a number of health related behaviours and choices [7-9]. Two studies showed that the association between patient activation and measures of health literacy was weak, indicating that these are two distinct concepts [7,8]. The other study distinguished between numeracy, literacy and activation [9]. In this study more activated patients were better able to understand and use comparative health care information, even when they had lower numeracy and literacy skill levels. It was suggested that activation might be a proxy for motivation and can compensate for lower skills. In all three studies, however, health literacy was defined on a functional level. The broader definition of health literacy by Nutbeam [10] distinguishes between functional, communicative/interactive and critical literacy. These latter components definitely show conceptual overlap with patient activation. Nutbeam includes aspects such as motivation, personal skills and self-efficacy in his definition of health literacy and acknowledges that ‘different levels of literacy progressively allow for greater autonomy and personal empowerment’ [10].

Knowing a person’s activation level is relevant because it can help providers to effectively communicate with their patients and to tailor health messages and self-management goals [4]. Compared to the regular patient approach, an intervention with tailored messages has proven to lead to greater improvement in the patients’ biometrical clinical indicators, in their adherence to prescribed medication regimens and to a reduction in hospitalizations and use of the emergency department [11]. Furthermore, patient activation has proven to be a changeable characteristic [6,12]. This makes the concept even more relevant since it can not only be used for categorizing patients and consumers and tailoring support and education, but also for actual improvement of consumer participation with respect to health and health care, both on an individual and on a population level.

The Patient Activation Measure has been studied extensively in the USA. Since many of the American problems regarding the management of health and chronic diseases also exist in the Netherlands, there is a definite clinical and scientific need for an instrument that can help differentiate patients and consumers into subgroups that require different strategies in health support, information and communication. An official, validated Dutch version of the PAM would be suitable and relevant for this purpose. Similar initiatives have been taken in Norway and Denmark [13,14]. Therefore, a research project was performed in the Netherlands in order to:

▪ translate the American short form Patient Activation Measure (PAM 13) into a Dutch version;

▪ establish the psychometric properties of the Dutch version of the PAM 13; and

▪ validate the Dutch version of the PAM in a panel of chronically ill patients.

For validation purposes, the concept of health literacy was chosen, even though previous studies have shown a weak association between the two [7-9]. Since all these studies were done in the USA, and the Netherlands’ health care system and patients are different to a major extent, the choice for replication was made. Only very recently, health literacy instruments were translated in Dutch [15] and available for clinical practice and research. Therefore this is the first opportunity to confirm or reject earlier findings on the relationship between patient activation and health literacy in the Dutch context.

## Methods

### Translation and adaptation process

For the translation and adaptation of the PAM 13, a systematic approach conform instructions of the WHO was followed [16]. This method includes the following steps: forward translation, expert panel meeting, backward translation, pre-testing/cognitive interviewing and consensus about the final version. Two independent Dutch translators performed the forward translation. The expert panel included both translators and three researchers with an expertise in chronic care, diversity in patient groups and measurement development. Discrepancies between the two translations were discussed and resolved. At the end of the expert meeting a single translation of the PAM 13 was agreed upon. This instrument was then translated back into English. There were only a few minor textual discrepancies between the backward translation and the original instrument, which led to the conclusion that the Dutch translation was satisfactory and ready for pre-testing.

Pre-testing was done in a focus group and 11 individual cognitive interviews. A sample of 185 former participants of the Dutch National Panel of people with Chronic illness or Disability (NPCD) were asked to participate in the pre-test. Five agreed to take part in the focus group (3 male, 2 female, mean age 64), whereas 11 preferred an interview (5 male, 6 female, mean age 65). Former participants were chosen because a sample from the current members of the Panel would be drawn for the main study (see under participants). In both the focus group and the interviews, respondents filled out the instrument at the beginning of the session. Then, the moderator (JR) or interviewer (JN) would ask questions about the general comprehensiveness of the instrument. Subsequently, each of the 13 items of the PAM was discussed. Respondents were asked to think out loud, contemplating about their interpretation and the possible meaning and phrasing of the item. Also the answering categories were discussed. The focus group and cognitive interviews didn’t lead to major alterations in the Dutch translation of the PAM 13, though some items were slightly rephrased. The final version of the instrument (PAM 13-Dutch) is attached as an Additional file 1 to this paper (copyright Insignia Health http://www.InsigniaHealth.com).

### Participants

The PAM 13-Dutch was sent to 2542 members of the Dutch National Panel of people with Chronic illness or Disability (NPCD) in April 2010 (study A) and again to 973 members in June 2010 (study B). The second sample were patients with Asthma and/or COPD, who had also received a questionnaire in study A. By doing so, the test-retest reliability of the instrument could be established.

The Dutch National Panel of people with Chronic illness or Disability (NPCD) is a nationwide prospective panel-study in the Netherlands. NPCD consists of over 4000 people aged 15 years and over with medically diagnosed chronic disease(s) and/or moderate to severe levels of physical disability. It has been set up to provide information with respect to the consequences of chronic illness and disability from the patient’s perspective. For the purpose of this study, we only selected panel members who had been diagnosed with a somatic chronic disease. Patients are included in the panel on the basis of a the oldest medical diagnosis of a chronic disease (=index disease). The medical diagnosis or diagnoses of chronic diseases of the patients were registered by their general practitioner (GP) using the International Classification of Primary Care (ICPC). The registered chronic disease (oldest diagnosis = index disease) of the participants in study A could be classified into nine broad categories: asthma and COPD (together 38%), diabetes (12%), cardiovascular diseases (11%), rheumatic diseases (11%), neurological diseases (6%), cancer (4%), chronic digestive diseases (4%) en other chronic diseases (15%). Asthma and COPD patients were oversampled in study A because they would be approached again for study B.

We had chosen to validate the PAM 13-Dutch in the NPCD subsample of people with a chronic illness because it closely resembled the samples which were used in the validation studies in the United States and Denmark [2,14]. In the USA, a group of 1515 randomly selected adults (age 45+) participated in the development study, of which 79% had at least one chronic disease [2]. In the Danish study, 467 patients (age 43 - 75 years) who were diagnosed with different aspects of dysglycaemia (Impaired Fasting Glucose, Impaired Glucose Tolerance and Type 2 diabetes) participated [14].

In both data collections, the instrument was part of a larger survey on chronic care and self-management. When entering the Panel, the participants give informed consent to the use of their data for research purposes. According to Dutch law, no further ethical approval is required.

### Measures

Age, gender, education level and self-reported health were measured as background variables. Education in the Netherlands was divided in three groups; low (no, primary school, or vocational training), middle (secondary or vocational education) or high (professional higher education or university). Self-reported health is the first item of the SF 36 (http://www.sf-36.org).

Patient activation was measured using the PAM 13-Dutch, which consists of 13 items. The answering categories per item are 4-point Likert scales, ranging from totally disagree to totally agree and ‘non applicable’. The design of the instrument reflects the four stages of activation in a progressing difficulty of the items: level 1 (patients believe that their role is important; items 1 and 2), level 2 (patients have confidence and knowledge to take action; items 3-8), level 3 (taking action; items 9-11) and level 4 (staying on course under stress; items 12 and 13) [2].

To establish the level of Health Literacy, we used the Dutch translation of the Set of Brief Screening Questions (SBSQ-D) and followed the corresponding scoring instructions [15]. This is a self report instrument which consists of three items and focuses on the ability to read and understand medical information. The Dutch version of the Set of Brief Screening Questions (SBSQ-D) of functional health literacy included: 1) “How often do you have someone help you read materials?”, 2) “How confident are you filling out medical forms by yourself?” and 3) “How often do you have problems learning about your medical condition because of difficulty understanding written information?” Question 1) and 3) had response options *neveroccasionallysometimesoften* or *always*, whereas for question 2) they were e*xtremely, quite a bit,somewhat, a little bit* and *not at all.* Answers were scored on a Likert scale from 1 to 4 where 1 was assigned to *never* or *extremely*, and 4 to merged responses *often/always* and *a little bit/not at all*. Cronbach’s α for the SBSQ-D was 0.73, indicating acceptable internal consistency.

### Statistical analyses

The analyses were performed on the data of study A, except for the test-retest reliability where the datasets from study A and B were combined. For comparability with the Dutch and American studies, we focused in our analyses on the subsample in study A of people with a chronic illness.

To establish the psychometric properties of the PAM 13-Dutch the data quality and internal consistency of the instrument were assessed. For data quality, we looked at the mean (with standard deviation), median, percentage of missing data, and percentage of ‘non applicable’ answers. Internal consistency was measured as the Cronbach’s α, inter-item and item-rest correlations (Pearson’s r). Item-rest correlations are the correlations between an item and the scale that is formed by all other items. Since very strong correlations are rare in the social sciences, in our study a correlation ≥ 0.50 is considered strong, r ≥ 0.30 moderate and r ≥ 0.10 weak [17]. Furthermore, the distribution of levels of activation and adjusted mean overall scores in the Dutch sample were described (by gender, age, educational level and self-reported health) and the latter were compared to the Danish and American scores.

To validate the PAM 13-Dutch, the test-retest reliability as an indicator of stability and construct validity was established. We also looked at associations between the PAM scores and a measure of Health Literacy to establish the construct validity. Analyses were performed with STATA 10.

## Results

### Participants

#### Study A

In study A 2542 questionnaires were sent of which 2070 were completed and returned. The gross response was therefore 2070/2542*100 = 81.4%. Of this sample, the data of 233 respondents were excluded: 198 because they had filled out less than 7 items on the PAM-Dutch questionnaire and 35 because they had responded to all the items with totally agree or disagree. These are requirements to be able to be able to compute a valid PAM-score. The net response rate in study A was therefore 1837/2542*100 = 72.3%. The mean age of these participants was 58.7 years (SD 0.38) (age 15 - 93 years). Of the sample, 56.2% was female. Asthma, COPD and diabetes were the most frequent reported chronic illnesses with a prevalence in the sample of respectively 20.3, 13.5 and 11.5 percent.

#### Study B

In study B 973 questionnaires were sent to patients with Asthma and/or COPD, who had also participated in study A. Of these questionnaires 705 were completed and returned (gross response: 705/973*100 = 72.5%). Of these, 33 respondents were excluded because they had filled out less than 7 items on the PAM-Dutch questionnaire and 26 because they had responded to all the items with totally agree or disagree. The net response for study B was: 672/973*100 = 69.1%. The mean age of these participants was: 58.7 years (SD 0.58) and 55.4% was female. The time between data collection of study A en B was approximately 8 to 12 weeks.

Of the remaining 672 respondents in study B, the data of 499 could be used for the test-retest analysis. The other 173 respondents had been excluded in study A because they had not filled out the PAM according to the requirements.

### Psychometric properties

To establish the psychometric properties of the PAM 13-Dutch the data quality, internal consistency and item-rest correlations were assessed (Table 2).

**Table 2:** Data quality and item-rest correlations of the Dutch 13-item PAM (N = 1837)

**Item**	**N**	**Mean PAM score**	**SD**	**Median**	**% missing values**	**% not applicable**	**Item-rest correlation**
1	1788	3.32	0.71	3	1.0	1.7	0.49
2	1542	3.20	0.69	3	3.3	12.8	0.50
3	1716	3.10	0.73	3	1.5	5.1	0.60
4	1736	3.20	0.66	3	0.4	5.1	0.54
5	1815	3.24	0.60	3	0.5	0.7	0.62
6	1805	3.29	0.61	3	0.9	0.9	0.55
7	1487	3.30	0.57	3	1.6	17.5	0.62
8	1771	3.23	0.67	3	0.9	2.7	0.66
9	1733	3.10	0.67	3	1.4	4.3	0.64
10	1565	3.04	0.66	3	1.7	13.1	0.51
11	1694	2.96	0.70	3	1.0	6.8	0.61
12	1667	2.62	0.75	3	1.1	8.1	0.50
13	1678	2.84	0.70	3	1.0	7.6	0.46

With respect to data quality, we looked at the mean (with standard deviation), median, percentage of missing data, and percentage of ‘non applicable’ answers. Overall, the mean scores on the items varied between 2.62 and 3.32. In general, the last items have a lower mean score compared to the earlier items in the questionnaire. However, in our results the individual item sequence did not exactly follow the original US scale (from higher to lower scoring items).

The item response is high, few missing values are reported (between 0.4% and 3.3%). Three items (7, 10 and 2) are scored as ‘not applicable’ by more than 10% of the participants. For item 7 (‘I am confident that I can follow through on medical treatments I need to do at home’) and item 10 (‘I have been able to maintain the lifestyle changes for my health that I have made’) this may actually be the correct answer. Item 2 (‘Taking an active role in my own health care is the most important factor in determining my health and ability to function’) requires rephrasing in the Dutch version: it has the highest percentage (3.3%) of missing values and furthermore 12.8% scored the item as not applicable, which is inappropriate since it is a general statement which applies to all participants.

Internal consistency was measured as the Cronbach’s α and inter-item correlations. Cronbach’s α for the sum scale was 0.88, which is similar to the Danish version (0.89) and is considered as a good level of internal consistency. The inter-item correlations ranged from 0.14-0.63. Item-rest correlations per item to the sum scale were moderate (for item 1 and 13) to strong (for all other items) and varied between 0.46 and 0.66.

The distribution of levels of activation and adjusted mean overall scores in the Dutch sample were described by gender, age, educational level and self-reported health. These data were compared to the Danish and American scores (Table 3). In the Dutch sample, there is a clear association between gender, age, educational level, self-reported health and patient activation score. In general male, younger, higher educated and healthier participants have higher scores on the PAM 13-Dutch. Self-reported health is the most distinguishing variable in this respect: whereas almost two thirds (64.6%) of the persons who report poor health have activation level 1 or 2, most people who report to be in good or very good health have activation level 3 or 4.

**Table 3:** Reliability of the Dutch 13-item PAM compared to the Danish and American version

			**PAM13**			**95% CI**	**PAM13 Danish**	**PAM13 USA**
	**Level 1**	**Level 2**	**Level 3**	**Level 4**	**Overall**		**Overall**	**Overall**
Sample (N)	314	359	590	574	1837		344	454
Mean activation score	41.1	50.3	59.9	79.7	61.3	60.6-62.0	64.2	61.9
Gender								
Male (N = 804)	12.6%	19.8%	32.6%	35.1%	62.9	61.8-63.9	63.8	60.2
Female (N = 1033)	20.6%	19.4%	31.8%	28.3%	60.0	59.1-61.0	64.7	62.8
Age groups (years)								
-54 (N = 625)	19.2%	18.1%	28.0%	34.7%	61.5	60.3-62.8	62.5	63.9^1^
55-64 (N = 476)	17.0%	17.2%	31.5%	34.2%	62.4	60.9-63.8	63.9	61.7
65-74 (N = 428)	13.1%	25.0%	35.1%	26.9%	60.5	59.1-61.9	65.6	61.9
75+ (N = 308)	18.5%	18.5%	37.3%	25.7%	60.0	58.3-61.7	56.6	58.2^1^
Educational level								
Low (N = 544)	19.5%	22.1%	36.0%	22.4%	58.4^2^	57.2-59.7	65.0^3^	58.5^4^
Middle (N = 731)	17.7%	18.7%	28.3%	35.3%	62.3^2^	61.1-63.5	62.8^3^	61.8^4^
High (N = 430)	12.8%	18.4%	34.7%	34.2%	62.5^2^	61.1-63.9	65.4^3^	61.6^4^
Self-reported health								
Excellent (N = 36)	5.6%	8.3%	25.0%	61.1%	74.2	68.2-80.2	76.3	68.7
Very good (N = 195)	4.1%	9.7%	30.8%	55.4%	71.6	69.4-73.9	66.6	64.3
Good (N = 1010)	12.4%	19.4%	34.8%	33.5%	62.3	61.4-63.2	62.6	59.3
Fair (N = 525)	28.8%	23.2%	29.5%	18.5%	55.8	54.4-57.0	62.5	57.3
Poor (N = 65)	36.9%	27.7%	21.5%	13.9%	52.6	49.4-55.8	57.4	54.3

Only questionnaires with at least 7 items answered were included

^1^ Extreme age group was 45-54 and 75-84.

^2^ Education in the Netherlands was divided in three groups; low (no, primary school, or vocational training), middle (secondary or vocational education) or high (professional higher education or university).

^3^ Education in Danish analyses was categorised as: unskilled, short (1-3 years) and higher (>3 years).

^4^ Education in American analyses was categorised as: high school or less, some college and college graduate+.

Comparing the Dutch results to the Danish and American data, we see that the Dutch mean PAM score (61.3) is comparable to the American (61.9) but somewhat lower than the Danish (64.2). While in the other countries women score higher on the PAM-13 than men, in the Netherlands the opposite is true. Associations with age, educational level and self-reported health in the three countries are similar. Also in Denmark and the USA, the association with self-reported health is strongest.

### Validation

To validate the PAM 13-Dutch, the test-retest reliability was established (Table 4). On item level, the correlations between the first and the second measurement varied between 0.25 and 0.49 (p < .001), and was moderate on average. For the scale as a whole, the correlation was 0.47 (p < .001).

**Table 4:** Test - retest reliability of the PAM between study A and study B

**Item**	**N**	**Mean PAM score (study A)**	**Mean PAM score (study B)**	**Pearson’s r**	**P-value**
1	475	3.31	3.30	0.35	<0.001
2	357	3.23	3.17	0.25	<0.001
3	448	3.12	3.09	0.31	<0.001
4	471	3.24	3.22	0.36	<0.001
5	490	3.22	3.25	0.32	<0.001
6	484	3.30	3.33	0.42	<0.001
7	393	3.36	3.43	0.41	<0.001
8	482	3.33	3.31	0.32	<0.001
9	467	3.11	3.03	0.42	<0.001
10	383	3.04	2.94	0.44	<0.001
11	448	2.98	2.90	0.40	<0.001
12	415	2.62	2.59	0.47	<0.001
13	427	2.83	2.83	0.49	<0.001
Total score	499	61.94	60.48	0.47	<0.001

To establish construct validity, correlations between the PAM scores and the SBSQ-D as a measure of Health literacy were computed. In our sample 23 respondents scored low on Health literacy (score of 2 or less on the SBSQ-D [15]), their mean PAM score was 48.6. Most participants (N = 1814) scored adequate on the SBSQ-D (score higher than 2). Their mean PAM score was much higher (61.4) compared to the low literacy group, indicating that as health literacy increases the activation level of patients generally goes up. The overall correlation between the SBSQ-D and PAM scores however was weak to moderate (r = 0.28).

## Discussion

The PAM-13 Dutch is a reliable instrument to measure patient activation. The level of internal consistency is good (α = 0.88) and item-rest correlations are moderate to strong. Compared to the Danish data, the internal consistency as measured by Cronbach’s α is comparable in the Dutch sample. Unfortunately, there is no α published for the American sample. Internal consistency might further be improved by rephrasing item 2 (‘Taking an active role in my own health care is the most important factor in determining my health and ability to function’) which has the highest percentage of missing values, a high and inappropriate level of ‘non applicable’ scores and a relatively low item-rest correlation. Though the individual item sequence in our study did not exactly follow the original US scale, in general the progressing difficulty of the items is reflected in the mean scores. Further validation in other Dutch samples and more specific statistical (Rasch) analyses will have to confirm whether the sequence of the original US scale will require readjustment in the Netherlands.

The stability and construct validity of the PAM-13 Dutch was measured by establishing the test-retest reliability and cross-validation with the scores on the SBSQ-D, a self report measure of Health literacy. The test-retest reliability was moderate. As in earlier studies in the USA [7-9] the association between the PAM and Health literacy in this study in the Netherlands was weak to moderate. Given the fact that the SBSQ-D only measures functional Health literacy, the lack of correlation in our study is understandable since patient activation is a broader theoretical concept. Unfortunately, as in the United States, validated and feasible self report instruments that measure Health literacy in a more comprehensive way, including communicative/interactive and critical skills, are not available in the Netherlands [15].

The Dutch mean PAM score (61.3) is comparable to the American (61.9) but lower than the Danish (64.2). This may be due to cultural differences. However, since Denmark and its social and health care system resembles the Netherlands more than the United States do, it would have been likely that contrasts would be most between the Netherlands and the United States. Though in the Netherlands patient choice and empowerment have become important themes in the past decade, the United States have a longer tradition in patient involvement and focussing on individual responsibilities, also with respect to health and health care. Since the difference with the Danish sample is the greatest, sampling differences will probably play a role as well. Further studies with comparable samples (e.g. by age, type of chronic disease) are necessary to be able to make more substantiated comparisons between countries.

Self-reported health is the most distinguishing variable with respect to the patient activation scores, both in the Netherlands as in Denmark and the United States. It is not possible in this cross-sectional study to establish cause and effect. It is known that people with low activation scores (level 1 and 2) are more often caught in a circle of negative emotions and low self esteem [3]. This might also influence a more negative assessment of their health status. However, since American studies have demonstrated that a higher score on the PAM positively influences various health related behaviours [3,4], one can expect a positive influence on health by improving the PAM and by helping professionals to tailor their care to the specific needs and possibilities of their patients, which may vary by their activation status. Further studies in this domain in the Netherlands, using the PAM-13 Dutch, are now beginning.

## Conclusions

The PAM-13 Dutch is a reliable instrument to measure patient activation. The level of internal consistency is good (α = 0.88) and item-rest correlations are moderate to strong. The test-retest reliability of the instrument was moderate. Cross-validation with the SBSQ-D, a self report measure on Health literacy, was weak to moderate. More research is needed into the validity of the Patient Activation Measure, especially with respect to a more comprehensive measure of Health literacy.

## Competing interests

The authors declare that they have no competing interests.

## Authors’ contributions

JR has contributed with the original idea for the questionnaire translation and the study design. She conducted the focus group (together with JN) and wrote the first draft of the manuscript. JN guided the translation process, conducted the cognitive interviews and performed qualitative and statistical data analysis. LvdH contributed to the general statistical data analyses. MH and MR were responsible for the data collection in the National Panel and assisted in drafting the manuscript. JR, JN and MR participated in the expert panel. All authors read and approved the final manuscript.

## Pre-publication history

The pre-publication history for this paper can be accessed here:

http://www.biomedcentral.com/1471-2458/12/577/prepub

## Supplementary Material

Additional file 1 Dutch version of the PAM-13 (PAM-13-Dutch). Click here for file
