# Supplementary material for: Measuring patient activation in the Netherlands: translation and validation of the American short form Patient Activation Measure (PAM13)
Source: BMC Public Health. 2012 Jul 31;12:577. doi: 10.1186/1471-2458-12-577 (PMC3490810; doi:10.1186/1471-2458-12-577)
Supplement: Additional file 1 — Dutch version of the PAM-13 (PAM-13-Dutch). [file 1471-2458-12-577-S1.doc]

**Patiënt Activatie Meetinstrument** (PAM-13 NL)

Hieronder staan enkele uitspraken die mensen soms doen over hun gezondheid. Geef voor elke uitspraak aan, in hoeverre u het ermee eens of oneens bent. Doe dit door het antwoord te omcirkelen dat het meest op uw persoonlijke situatie van toepassing is. *We willen dus weten wat u zélf vindt en niet wat u denkt dat de dokter of onderzoeker wil horen.*

Als de uitspraak niet op u van toepassing is, omcirkel dan ‘n.v.t.’

| 1. | Uiteindelijk ben ik zelf verantwoordelijk voor mijn gezondheid. | Helemaal niet mee eens | Niet mee eens | Mee eens | Helemaal  mee eens | n.v.t. |
| --- | --- | --- | --- | --- | --- | --- |
| 2. | Een actieve rol op me nemen in de zorg voor mijn gezondheid, heeft de meeste invloed op mijn gezondheid. | Helemaal niet mee eens | Niet mee eens | Mee eens | Helemaal  mee eens | n.v.t. |
| 3. | Ik heb er vertrouwen in dat ik kan bijdragen aan het voorkomen of verminderen van problemen met mijn gezondheid. | Helemaal niet mee eens | Niet mee eens | Mee eens | Helemaal  mee eens | n.v.t. |
| 4. | Ik weet wat elk van mijn voorgeschreven medicijnen doet. | Helemaal niet mee eens | Niet mee eens | Mee eens | Helemaal  mee eens | n.v.t. |
| 5. | Ik heb er vertrouwen in dat ik kan beoordelen of ik naar de dokter moet gaan of dat ik een gezondheidsprobleem zelf kan aanpakken. | Helemaal niet mee eens | Niet mee eens | Mee eens | Helemaal  mee eens | n.v.t. |
| 6. | Ik heb er vertrouwen in dat ik een dokter mijn zorgen durf te vertellen, zelfs als hij of zij daar niet naar vraagt. | Helemaal niet mee eens | Niet mee eens | Mee eens | Helemaal  mee eens | n.v.t. |
| 7. | Ik heb er vertrouwen in dat het mij lukt om medische behandelingen die ik thuis moet doen uit te voeren. | Helemaal niet mee eens | Niet mee eens | Mee eens | Helemaal  mee eens | n.v.t. |
| 8. | Ik begrijp mijn gezondheidsproblemen en wat de oorzaken ervan zijn. | Helemaal niet mee eens | Niet mee eens | Mee eens | Helemaal  mee eens | n.v.t. |
| 9. | Ik weet welke behandelingen er zijn voor mijn gezondheidsproblemen. | Helemaal niet mee eens | Niet mee eens | Mee eens | Helemaal  mee eens | n.v.t. |
| 10. | Ik heb veranderingen in mijn leefstijl (zoals gezond eten of bewegen) kunnen volhouden. | Helemaal niet mee eens | Niet mee eens | Mee eens | Helemaal  mee eens | n.v.t. |
| 11. | Ik weet hoe ik gezondheidsproblemen kan voorkomen. | Helemaal niet mee eens | Niet mee eens | Mee eens | Helemaal  mee eens | n.v.t. |
| 12. | Ik heb er vertrouwen in dat ik zelf oplossingen kan bedenken voor nieuwe problemen met mijn gezondheid. | Helemaal niet mee eens | Niet mee eens | Mee eens | Helemaal  mee eens | n.v.t. |
| 13. | Ik heb er vertrouwen in dat ik veranderingen in mijn leefstijl (zoals gezond eten en bewegen) kan volhouden, zelfs in tijden van stress. | Helemaal niet mee eens | Niet mee eens | Mee eens | Helemaal  mee eens | n.v.t. |
